# Remote Sensing of Ecosystem Health: Opportunities, Challenges, and Future Perspectives

**DOI:** 10.3390/s141121117

**Published:** 2014-11-07

**Authors:** Zhaoqin Li, Dandan Xu, Xulin Guo

**Affiliations:** Department of Geography and Planning, University of Saskatchewan, 117 Science Place, Saskatoon, SK S7N 5C8, Canada; E-Mails: zhl237@mail.usask.ca (Z.L.); dax890@mail.usask.ca (D.X.)

**Keywords:** ecosystem health assessment, optical remote sensing, radar, LiDAR, ecosystem vigor, ecosystem organization, ecosystem resilience

## Abstract

Maintaining a healthy ecosystem is essential for maximizing sustainable ecological services of the best quality to human beings. Ecological and conservation research has provided a strong scientific background on identifying ecological health indicators and correspondingly making effective conservation plans. At the same time, ecologists have asserted a strong need for spatially explicit and temporally effective ecosystem health assessments based on remote sensing data. Currently, remote sensing of ecosystem health is only based on one ecosystem attribute: vigor, organization, or resilience. However, an effective ecosystem health assessment should be a comprehensive and dynamic measurement of the three attributes. This paper reviews opportunities of remote sensing, including optical, radar, and LiDAR, for directly estimating indicators of the three ecosystem attributes, discusses the main challenges to develop a remote sensing-based spatially-explicit comprehensive ecosystem health system, and provides some future perspectives. The main challenges to develop a remote sensing-based spatially-explicit comprehensive ecosystem health system are: (1) scale issue; (2) transportability issue; (3) data availability; and (4) uncertainties in health indicators estimated from remote sensing data. However, the Radarsat-2 constellation, upcoming new optical sensors on Worldview-3 and Sentinel-2 satellites, and improved technologies for the acquisition and processing of hyperspectral, multi-angle optical, radar, and LiDAR data and multi-sensoral data fusion may partly address the current challenges.

## 1. Introduction

Nature supplies food and recreation that human beings rely on. Unfortunately, ecosystems worldwide are threatened by anthropological activities and climate change [1]. Under such pressures, maintaining a healthy ecosystem is essential for supplying stable and sustainable goods and services for human societies [2]. Assessing and monitoring ecosystem health can not only provide early warning of environmental degradation, but also identify the cause of an existing problem [3]. It thus is an important and initial step for ecological conservation and ecological service assessment.

Ecosystem health assessment (EHA) as a part of environmental management that began in the late 1980s. Ecosystem health merged the concept of ecosystem with health science [4] and integrated social and physical science [5]. The early definition of ecosystem health was simply animal health or plant health [6]. However, the definition of ecosystem health should consider the ecosystem as a complex system to emphasize the connection between community process and physical environment [7]. Early ecosystem health research evaluated ecosystem health using keystone species [8]. However, keystone species evaluation cannot present the energy flux, nutrient cycle, productivity, diversity or response capacity to disturbance, although it may indirectly present the interaction among keystone species, other species, or the physical environment in the ecosystem. In 1999, Costanza and Mageau defined ecosystem health as “a comprehensive, multi-scale, dynamic, hierarchical measure of system resilience, organization, and vigor” [9]. Based on this definition, health condition of one specific ecosystem can be assessed by measuring the integrated ecosystem attributes: vigor, organization, and resilience [9], although it is impossible to set up a number of specific health indictors for all ecosystems to assess health status [10].

Traditionally EHA was conducted based on the field ecological data and (or) models driven by such field data, which cannot be widely applied at a large spatial scale [11] and has difficulty in providing spatially and temporally explicit assessment [12]. Nevertheless, there is an urgent need to understand and monitor the spatial heterogeneity of ecosystem health [13] for the purpose of optimum conversation [14].

Remote sensing data have potential for assessing and monitoring ecosystem health at different temporal and spatial scales across extensive areas with a broad extent [12,15]. They can be used for directly detailing ecological health indictors, such as productivity, species richness, and resilience after natural and human-induced disturbances [12] and for indirectly providing inputs for spatially explicit ecological process modeling [16]. To date, the application of remote sensing on EHA or monitoring has been focused on single ecosystem attribute, such as vigor (productivity [17–19], or species invasion [20,21]) or resilience (response to stress (fire [22], or climate change [23,24])). These studies are beneficial to relevant ecological studies using remote sensing in terms of their methods and conclusions. However, it is impossible to understand a complex ecosystem through only one ecosystem attribute [9]. A comprehensive and dynamic ecosystem health assessment with the integration of ecosystem vigor, organization, and resilience is urgently needed. Establishing such a spatially explicit EHA and monitoring system needs the close collaboration of both remote sensing specialists and ecologists [25].

Reviews have surveyed remote sensing of land cover, biodiversity, and carbon flux related, water flux related, and soil-based ecosystem services [26,27]. There are also summaries on the role of remote sensing in ecological applications [12,28]. However, to our knowledge, none of the reviews has focused on the opportunities and challenges of developing a comprehensive remote sensing-based spatially explicit EHA and monitoring system.

The objective of this review is to survey opportunities and challenges, and to provide future perspectives of using remote sensing for a comprehensive, spatially and temporally explicit dynamic EHA. The second section briefly introduces a framework for developing a spatially dynamic EHA system with the involvement of remote sensing specialists and ecologists. The third section summarizes a number of studies and reviews on predicting ecosystem health indicators of the ecosystem attributes: vigor, organization and resilience using remote sensing imagery. In some ecosystems, such as semiarid grasslands, Non-Photosynthetic Vegetation (NPV) biomass and Biological Soil Crust (BSC) are also important indicators of ecosystem vigor. Therefore, a detailed review on NPV cover/biomass and BSC cover is also included in this section. The fourth section focuses on current challenges of remote sensing of ecosystem health with detailed discussion on the effects of NPV, BSC, and bare soil on application of optical remote sensing in sparsely vegetated ecosystems. Finally, the fifth section summarizes current opportunities and challenges of remote sensing of ecosystem health and discusses the upcoming new opportunities. Our efforts are to summarize and discuss how and what remote sensing can contribute to EHA, leaving selections of ecological indicators out of current scope. A detailed introduction on how to select ecological indicators can be found in [10].

## 2. A Framework of a Remote Sensing-Based Ecosystem Health Assessment

The spatially explicit nature of remote sensing data with frequent revisit cycles provides an opportunity to assess and monitor the spatial heterogeneity of ecosystem health. Nonetheless, concerns were raised that remote sensing specialists may pay more attention to technology than ecological problems [29,30], while ecologists may not have sufficient remote sensing background to address ecological problems at local to global scales [25]. Efforts thus are needed to bridge the research gap between the two research communities.

**Figure 1.**
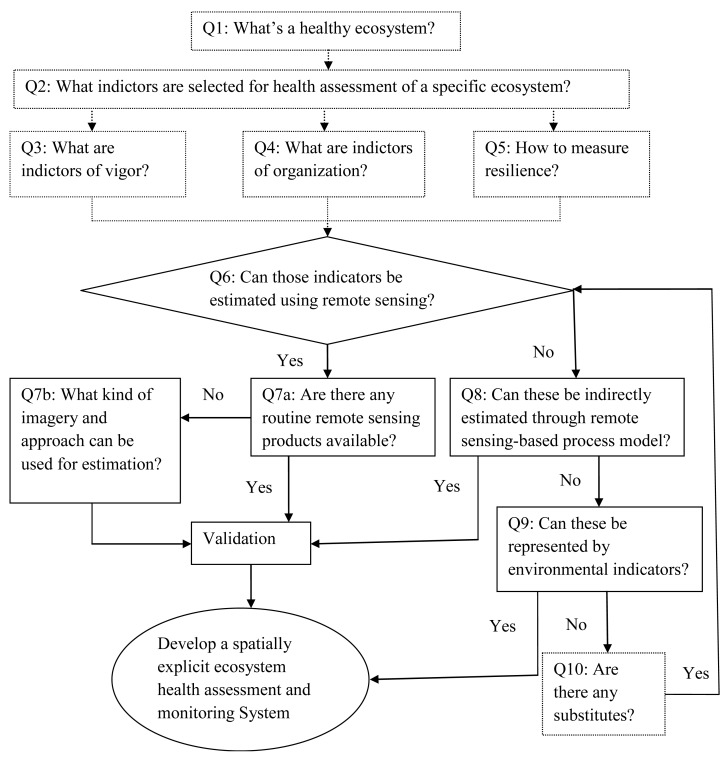
Procedures to integrate the expertise of remote sensing experts and ecologists to develop a remote sensing-based Ecosystem Health Assessment and Monitoring System. The questions outlined in dotted lines shows the contribution of ecologists.

To develop a comprehensive remote sensing-based EHA system, one might follow the procedures proposed in Figure 1 with the participation of remote sensing experts and ecologists. The cooperation of the experts in both remote sensing and ecology fields allow the effective health indicators to be identified efficiently and make sure that those indicators can be measured by remote sensing data.

Although Figure 1 includes indirect estimation of health indicators through modeling using remote sensing data as input, this review focuses on the questions we proposed: Q7a: Are there any routine remote sensing products for health indicators? Q7b: What kind of imagery and approach can be used to estimate health indicators, and challenges and future opportunities to develop a remote sensing- based spatially explicit EHA and monitoring system (the ultimate goal presented in Figure 1).

## 3. Remote Sensing of Ecosystem Health

Dynamic and integrated measurements of ecosystem attributes (vigor, organization, and resilience) allow an effective ecosystem health assessment and monitoring. For each ecosystem attribute, there are a number of indicators, although those indicators may be different for different ecosystems. This section will review the potential of remote sensing for estimating indicators of the three ecosystem attributes.

### 3.1. Remote Sensing of Vigor

Vigor can be measured through metabolism, yield, and soil fertility [31]. The most commonly used vigor indicator is Net Primary Productivity (NPP) or Gross Primary Production (GPP) of an ecosystem [9,32]. Other indicators that are directly or indirectly associated with NPP are green vegetation cover, green vegetation biomass, NPV cover or biomass, green ratio (green/dead vegetation cover or biomass), bare soil cover and BSC cover in semiarid and arid regions, and vegetation biological properties (chlorophyll, nitrogen, phosphorous, and moisture content *et al.*). In addition, increase in NPP sometimes does not mean an improved ecosystem if the increase is attributed to expansion of invasive plant species [32]. Therefore, distribution of invasive plant species is considered to be another potential indicator of ecosystem vigor.

#### 3.1.1. NPP or GPP

Changes in NPP are often used to evaluate environmental degradation in the context of desertification, impacts of pollution, climate change, and deforestation [33]. NPP can be estimated and monitored from optical remote sensing images since the 1970s [26], yet remote sensing derived daily global NPP products were not operationally produced until mid-2000s [34]. The modeling approach for predicting NPP is based on the light use efficiency (LUE) concept proposed by Monteith [35] and modified by Prince [36]. Based on the concept, the GPP of one ecosystem can be a function of the absorbed Photosynthetically Active Radiation (PAR) namely absorbed solar radiation at 400 to 700 nm wavelength and the photosynthetic efficiency which is specific for an individual plant type. NPP is the product of GPP by subtracting respiration. This LUE based modeling approach has been applied to produce Moderate Resolution Imaging Spectroradiometer (MODIS) global 8-day GPP and annual NPP at 1 km spatial resolution products (MOD17) [34], which have been ready for monitoring ecological conditions and environmental changes [37] since the mid-2000s.

For studies at regional or even smaller scales, statistical empirical model of GPP or NPP and a vegetation index, such as Normalized Difference Vegetation Index (NDVI), is more operational, considering the large number of parameters of the LUE model which highly affect the accuracy of GPP (or NPP) [26]. Good NDVI-GPP (or NPP) relationships have been observed in low biomass vegetated areas, such as the Arctic tundra [38] and the steppe [39]. However, NDVI becomes saturated at high biomass vegetated areas [40] including dense grass areas, forests, and croplands *etc.*, and thus result in significant difference in spatial distribution of NDVI and NPP [41]. The enhanced vegetation index (EVI) thus was developed for MODIS, and showed a good performance to overcome the saturation limitation of NDVI [42]. In addition, the accuracy of GPP estimation from the empirical relationship with vegetation indices is influenced by the spectral resolution of remote sensing data. For example, NDVI derived from EO-1 Hyperion and MODIS with higher spectral resolution yielded more accurate GPP estimation than Landsat ETM+ with lower spectral resolution in a mountainous meadow ecosystem [43].

#### 3.1.2. Green Vegetation, NPV, BSC, and Bare Soil Cover

The fractional cover of green vegetation, NPV, and bare soil can be estimated simultaneously using a spectral unmixing approach (SMA) (e.g., [44–46]), or using the empirical relationships between each cover and spectral indices (e.g., [47,48]). Green vegetation cover and bare soil estimation will not be discussed in depth here, as the former has been routinely produced as traditional remotely sensed products from MODIS, AVHRR, and SPOT-VGT *etc.* and the latter can be estimated together with NPV and green vegetation. NPV is a significant component of vegetation productivity in grasslands, savannas, shrublands, and dry woodlands [49] as well as wetlands [50]. BSC is present in semiarid and arid areas worldwide [51]. Both NPV and BSC are ecologically important, yet estimating their abundance using remote sensing appraoches are still very challenging. Thus they are our review focus in this subsection.

NPV can be separated from green vegetation in the visible (VIS, 400–700 nm) and near-infrared (NIR, 700–1200 nm) wavelength regions due to the lack of absorption of pigments [49], and from bare soil in shortwave infrared (SWIR, 1100–2500 nm) region due to the unique absorption features of lignin and cellulose of NPV [49,52]. Efforts have been made to estimate NPV cover using optical remote sensing data, especially hyperspectral images, in croplands (e.g., [47,53–55]), grassland [56], savannah [46], and forest ecosystems [44]. However, the estimation of NPV abundance is highly affected by soil mineralogy and soil organic carbon [57,58], moisture content of both green vegetation and bare soil [52,57], and the decomposition of NPV [52]. In this regard, radar and LiDAR remote sensing which have an advantage to detect the structure of NPV can be good data sources for NPV estimation. For detailed information on remote sensing of NPV abundance using optical, radar, and LiDAR data, please refer to [59].

The spectral characteristics of BSC have been investigated by many researchers (e.g., [60–63]) using field measurements. Although an absorption feature at approximately 680 nm has been always observed in BSC samples, there is noticeable difference in the spectra as dominant BSC species change [61,64]. A recent study found that water absorption feature at about 1450 nm can be used to differentiate BSC from green vegetation and the spectra of the most developed BSCs is characterized by a steeper slope between about 680 and 750 nm [65]. Based on the spectra characteristics, many efforts have been made to detect and map BSC using Landsat MSS, TM, or ETM+ images [61,64,66–68]. These studies have demonstrated that remote sensing has a great potential to detect and map spatial distribution of BSC on a large spatial extent timely and efficiently [67,69]. However, the variations in spectra of different BSC communities make the derived spectral indices less universally applicable for mapping BSC cover. The crust index developed for mapping cyanobacteria-dominated BSC [64] is not suitable for lichen-dominated BSC covering large areas of cool and cold deserts [70]. Another BSC index (BSCI) was proposed to discriminate lichen-dominated BSC from land surface of bare sand and dry plant material in a desert [67], however, the usage of BSCI was highly affected by the predetermined lower and upper thresholds of BSCI. Besides the crust indices, continuum removal [71], SMA approaches [72], and a partial least squares regression-linear discriminant analysis [64] were also used for BSC investigation. The conclusions of these studies are not always consistent for different study areas. For instance, hyperspectral images were thought not to be able to effectively differentiate BSCs when there was a mixed pixel with plants [73], while hyperspectral images with continuum removal crust identification algorithm (CRCIA) could work reliably for BSC identification in the presence of plant litter and plants [71], and [72] concluded that hyperspectral images can be used to monitor local or even regional changes of BSC in the southwestern deserts of the United States. Considering the inconsistent conclusion on the applicability of hyperspectral imagery, further research is needed in other arid and semiarid ecosystems, such as semiarid mixed grasslands.

#### 3.1.3. Vegetation Biochemical Properties

Vegetation biochemical properties, such as chlorophyll (Ch), nitrogen (N), and phosphorous (P) are highly related to ecosystem functioning, thus are important indicators of ecosystem health assessment [74]. Ch controls photosynthesis, and is thus an indicator of plant health and GPP [75]. P as an indicator of nutrient quality of plant and plant growth rate [74] can also be an indicator of plant health. N being an important component of Ch is also strongly associated with plant health and GPP. Remote sensing of vegetation biochemical properties have been successfully conducted at a leaf level for a few decades using narrow band spectral indices derived from ground and space hyperspectral data. For a list of hyperspectral sensors please refer to [59]. Efforts have been made to scale up biochemical content to canopy level using remote sensing data in crops and forests [76] and semiarid mixed grassland [77]. Methods used for scaling up biochemical contents from leaf to canopy level were summarized by [76]. Yet it is still challenging for biochemical content estimation at a landscape level, despite promising [76,78].

Due to the importance, Ch has drawn a particular attention of both ecologists and remote sensing scientists. Ch has been estimated using red edge position (REP) based on the finding that an increase in chlorophyll content will be reflected on the spectra as the wavelength edge of red absorption feature moves to even longer wavelengths [79]. However, REP cannot accurately estimate high Ch content [79,80]. In addition, spectral indices developed for chlorophyll estimation were summarized and compared by [81,82], and red-edge based vegetation index has demonstrated more potential for chlorophyll content estimation in a semiarid mixed grassland ecosystem of Canada [77]. The estimation of Ch mainly uses continuous wavelength ranges or narrow bands spectral indices. However, space sensed data with fine spectral resolution including the Medium Resolution Imaging Spectrometer (MERIS) and the upcoming new satellite sentinel-2 have also demonstrated great potential for Ch estimation [83,84]. Terrestrial chlorophyll index (MICI) was developed based on band 8, 9, and 10 of the MERIS, and global composites of MICI at 300 m spatial resolution, as a unique terrestrial chlorophyll product have been produced under the support of the European Space Agency (ESA) since 2006 [85].

There are a number of studies on remote sensing of vegetation N at leaf and canopy level with high accuracy [74], while only limited research on P estimation and the accuracy of estimation is lower than N estimation [86]. The commonly used approach for estimating these vegetation biochemical properties are empirical methods based on *in situ* measurements of biochemical content and remotely sensed data. The most widely used wavelengths for N and P estimation are the NIR and SWIR regions [86]. The estimation of these biochemical properties thus is highly influenced by canopy water content. To minimize water absorption effects and other influences from atmosphere, soil, and redundancy of hyperspectral data, spectral indices, first derivative, continuum removal, and log-transformed spectra have been used to boost the absorption features of vegetation biochemical properties. Water-removed spectra constructed based on a nonlinear combination of a dry-matter and a leaf water spectrum [87] increased the accuracy of N and P estimation of grass in savanna, compared to first derivative and continuum removal spectra [86]. The commonly used empirical models for predicting biochemical properties based on biochemical spectra features are simple linear regression, partial least-squares regression (PLSR), and stepwise multiple linear regression (SMLR). The spectral indices used for N estimation can be found in [88] but mainly for crops, while no VI was specifically designed for P estimation yet [74].

#### 3.1.4. Invasive Plant Species

Invasive plant species in diverse ecosystems can be shrubs, trees, and herb species, which alter biodiversity, structure, and function of ecosystems [89]. Identification of invasive tree and shrub species using remote sensing was successfully demonstrated [90–93] using multispectral medium spatial resolution Landsat, high spatial resolution IKONOS, or hyperspectral images. Remote sensing of herb species is much more difficult and highly dependent on the separation of the species from surrounding species and background [94]. Due to the difficulty, identification of herb species was mostly conducted using hyperspectral images (reviews of [89,95]). However, high spatial resolution IKONOS (4 m) with texture information [96] and very high spatial resolution aerial photography [94,97] also mapped herb invaders with high accuracy. Therefore, it should be cautious to select remote sensing data with suitable spatial and spectral resolution for specific species recognition (for review see [95]). Methods used for invasive species identification mainly include visual interpretation and pixel-based and object-oriented image classification [95]. The spatial resolution issue may be overcome through the methods include SMA of one pixel [92] and combination of other ancillary data. Spectra resolution limitations may be overcome by selecting appropriate period or using time series data to maximizing difference in spectra between invasive species, native species, and backgrounds.

### 3.2. Remote Sensing of Organization

Ecosystem organization represents both species diversity and the interactions between species within the system [98]. The indicators of organization can be species richness, landscape diversity, structural traits including canopy height, Leaf Area Index (LAI), canopy morphology, and horizontal structure represented by the spatial arrangement of green vegetation, NPV, and bare soil. Since remote sensing of green vegetation, NPV, and bare soil has been reviewed in last section, we will focus on species richness and biodiversity and structural traits here.

#### 3.2.1. Species Richness and Biodiversity

Species richness is a primary measurement of regional or community biodiversity [99]. Due to the ecological importance of biodiversity, considerable research and a few reviews [42,100–103] have been completed on remote sensing of species richness. To date, species richness studies have been conducted using one sensor imagery at specific time of periods [104], and more recently using images of multiple passive sensors over multiple time periods [105]. Generally remote sensing of species richness can be classified as direct mapping and indirect modeling approaches [100,101]. Direct mapping species distribution using remote sensing is similar to mapping invasive plant species in terms of approaches and potential and limitations of remote sensing data. Indirect modeling approaches have been widely used to predict species richness based on the empirical relationships between field species investigation and information derived from remote sensing, such as land cover and landscape metrics, NPP, and spectral variation [100,101].

The rationale on why land cover and landscape metrics can be correlated to species richness or biodiversity is that land cover and landscape metrics including fragmentation [12] have certain associations with species existence. Such land cover information has been used for predicting species richness [12,106]. This method may be suitable for species richness investigation at large spatial scales. However, prediction accuracy of species richness using such method is arguable due to three aspects: (1) environmental factors including temperature, precipitation, disturbance, and others were neglected [107]; (2) this method is highly affected by spatial resolution of remote sensing imagery [108]; and (3) the derived landscape-metrics do not contain internal information of the metrics [104].

The relationship between NPP and species richness was established based on the species-energy theory which hypothesizes that species richness is correlated with NPP [109]. Thus, NDVI with a close relationship with NPP [36] has been widely used to predict species richness [12,100]. The utilization of NDVI is mainly based on NDVI variation, and positive relationship between NDVI and specie richness was found in [105,110]. However, there are inconsistent conclusions that state little correlation between NDVI variation and species richness. Research indicated that NDVI variation has negative relationship with species richness [104] based on the hypothesis that low NDVI variation, higher homogeneity, and consequently higher species richness [111]. Although there are inconsistent conclusions, using NDVI for species richness prediction is still an effective method at large spatial scales [111].

The application of spectral variation for predicting species richness is based on spectral variation hypothesis (SVH) proposed by [112] which assumes that the higher variation in spectra, the higher heterogeneity of habitats allowing coexistence of more species, and consequently higher species richness [113]. In this domain, spectral indices, land-cover heterogeneity, and spectral variability derived from optical remote sensing data have been used to predict species richness [114]. SVH approaches will be an important direction of optical remote sensing of species richness [101].

Overall, the landscape metrics and NDVI approach are more suitable for species richness estimation at large spatial scales, while SVH can be used at a fine scale. However, prediction of species richness is affected by both spatial and spectral resolutions of satellite imagery [114]. For the SVH approach, multispectral imagery generally has difficulty in providing sufficient information for species richness prediction as it is hard to be used for retrieving biochemical and canopy structure information [115]. Hyperspectral images have an advantage as they can provide information on canopy biochemical elements including Ch, N, and cellulose content [116]. In addition, ancillary data, such as temperature, precipitation, and topography, can significantly contribute to species richness estimation [117].

#### 3.2.2. Structural Traits

Structural traits including canopy height, LAI, canopy morphology can be derived from optical remote sensing data through empirical relationships with vegetation indices or image texture metrics [118,119]. Global LAI products have been produced using MODIS and Cyclopes remote sensing data [120]. Nevertheless, more accurate estimation of these structural parameters can be achieved through LiDAR (e.g., [121,122]) and radar data (e.g., [27,123]). For example, global tree height has been mapped using the point samples of the spaceborne LiDAR GLAS data, and spatial continuity of tree height was achieved via MODIS reflectance data [124].

### 3.3. Remote Sensing of Resilience

Ecosystem resilience means an ecosystem's ability to remain in its current state and return to said state from stress [98], mainly consisting of natural climate change, wildfire, and anthropological activities, such as grazing and prescribed fire. Resilience at a given time may be assessed based on a ratio of a given ecosystem health indicator, such as aboveground biomass, measured between the post- and the pre-disturbance [125]. Remote sensing with a capability for ecosystem health indicator retrieval also provides an opportunity to estimate ecosystem resilience to disturbances. However, such remote sensing data should be able to be frequently acquired in a long time series to cover the regeneration time.

NDVI data have been widely used to evaluate ecosystem resilience to climate change (e.g., [126,127]), fire (e.g., [128–130]), and grazing (e.g., [131–133]), although other vegetation indices (e.g., Adjusted Transformed Soil-Adjusted Vegetation Index, ATSAVI) were also frequently used. Considering the requirement on time-series data, Landsat MSS, TM, ETM+, and OLI, MODIS, AVHRR, and SPOT-VEG NDVI data are normally options for resilience estimation. Nevertheless, using those NDVI data for evaluating resilience, precautions should be made to minimize the effects of seasonal and inter-annual variations of phenology and climate [128]. To minimize such effects, the quotient NDVI, the average NDVI measurements in the disturbed area divided by the average NDVI measurements in the surrounding undisturbed area, was calculated for resilience evaluation [128]. However, the surrounding undisturbed area should have similar vegetation, topography, and geology to the disturbed area [128]. In certain instances, although the surrounding undisturbed area can be a good reference, it is difficult to find suitable benchmarks [134]. Thus, a dynamic reference-cover method was proposed to separate grazing and rainfall effects in rangelands using remote sensing imagery [134].

## 4. Challenges to Developing a Remote Sensing Based EHA System

Remotely sensed data can be used to retrieve a variety of ecosystem health indicators as surveyed above. However, different indicators may require different remote sensing data for higher accuracy of estimation. Hence, there are multiple challenges to combining indicators of vigor, organization, and resilience for establishing a comprehensive, temporally and spatially explicit EHA system. In addition to the lack of a good solution to estimate NPV and BSC cover using remote sensing data as reviewed in Subsection 3.1.2, there are other challenges such as: (1) scale issue; (2) transportable difficulty; (3) data availability; and (4) uncertainties in retrieved ecosystem health indicators from remote sensing data.

### 4.1. Scale Issue

Species distribution and ecological processes are scale dependent on the growing conditions of species [135] partially controlled by soil and topography. The spatial scale issue has been identified as a major challenge in ecological assessment of remote sensing [135]. In part, the accuracy of the retrieval of vegetation properties using remote sensing depends upon sensor spatial resolution [136]. Using remote sensing data, especially low spatial resolution data, such as the 1 km spatial resolution Advanced Very High Resolution Radiometer (AVHRR), for ecosystem health assessment may introduce uncertainties resulting from land surface heterogeneity and mixed pixels containing more land cover types [137]. GPP calculated from the Region Production Efficiency Model (REG-PEM, [138]) with all model inputs obtained from AVHRR 1 km remote sensing data is significantly different from the GPP calculated using Landsat TM 30 m data [137]. However, finer spatial resolution remote sensing data cannot guarantee higher accuracy of ecosystem assessment [26]. For example, MODIS EVI at 250 m resolution cannot be used for estimating GPP of coniferous forests, while MODIS 1 km EVI can [42]. It was also found that when the spatial resolution of remote sensing data is higher than 60–80 m, the accuracy of forest classification decrease [139]. At the same time, a suitable spatial scale or satellite image at optimal spatial resolution can improve vegetation vertical structure (e.g., LAI) estimation in grassland because the land surface heterogeneity was minimized [140–142]. An optimum resolution can be detected using wavelet or semivariagram analysis, while considering a specific study goal.

In addition, some retrieval algorithms and models for ecosystem health indicators, such as biochemical properties, are derived at small scales for homogeneous land surfaces [143]. Applying those algorithms and models to large scales (typically imply heterogeneous land surface), may incur scale effects. Besides heterogeneity, the linearity or nonlinearity of retrieval models is the other factor on scale effects, and generally the former may result in smaller scale effects than the latter for mixed pixels with unknown mixture of different land covers [144]. Therefore, cautions should be made to select up-scaling approaches for the purpose of minimizing such scale effects. Some scaling methods have been summarized, although no universal scaling method was found [143]. More recently, a conceptual framework was proposed to scale up biochemical content in semi-arid mixed grassland from leaf to canopy level [76], and the estimation of grassland chlorophyll content at leaf, canopy, and landscape scales is fairly accurate [77].

Besides spatial resolution and scaling methods, the accuracy of ecological assessment using optical remote sensing also relies on spectral and temporal resolutions of sensors [135,136]. As discussed in Section 3, some ecological health indicators may be retrieved from remote sensing at high spatial resolution imagery, while others may need higher spectral resolution or temporal resolution data. In this regard, to develop a comprehensive ecosystem health assessment and monitoring system, data fusion can be a solution. The fused imagery can provide the maximum amount of useful information [145], and thus have significant advantages over each single source data [146].

### 4.2. Transportability Issue

The approaches used to retrieve health indicators from remotely sensed data are commonly empirical relationships between the predicted variables and reflectance (or spectral indices) of optical sensors, backscatter (or variables derived from backscatter, such as canopy water content and cross-polarized ratio) of radar, or LiDAR intensity. Indicators retrieved from empirical relationships can be difficult to transport to different sensors and study areas [27]. Nevertheless, efforts have been made to develop general models with promise in estimating foliar nitrogen [147] and biomass [121].

The other approaches used for retrieving health indicators from optical sensors are inversion of radiative transfer models and SMA approaches. Radiative transfer models provide better transportability for estimating health indicators, however; inversions of radiative transfer models are complex to implement with many input parameters and difficult to invert even if approaches, such as neutral network was applied [148]. SMA approaches are also more general to operate, while the temporal and spatial variability of end members may reduce the generality [149].

### 4.3. Data Availability

Ecosystem health indicators, such as biochemical properties and invasion species identification, require hyperspectral images, and others including canopy height and canopy morphology may need LiDAR data. The hyperspectral and LiDAR sensors usually are not activated until request. In addition, the imagery acquired has a very small footprint and consequently do not provide a global coverage, and such data are usually costly [150].

### 4.4. Uncertainties in Ecosystem Health Indicators

Uncertainties in the estimated ecosystem health indicators are one of the most important factors needed to be taken into account while developing an EHA system. There are still potential uncertainties on the estimation of indicators even if the most appropriate remotely sensed data are used.

#### 4.4.1. Optical Remote Sensing

**Figure 2.**
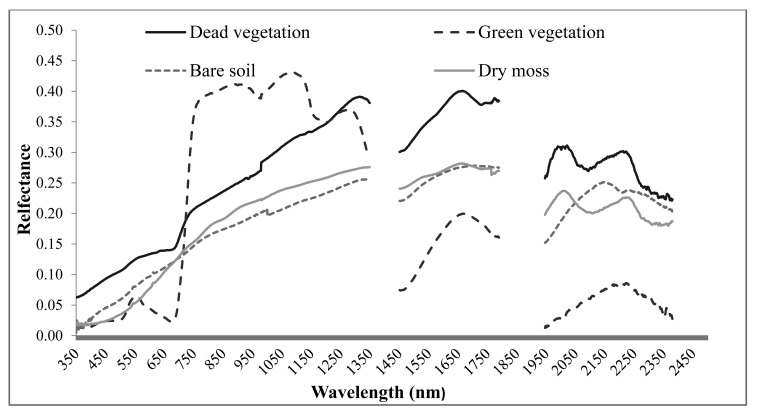
Spectral response curves of dead vegetation, green vegetation, bare soil, and dry moss as dominated BSC (samples were collected from Grasslands National Park, Canada and their spectra were measured in laboratory with an ASD Spectroradiometer).

Although selecting appropriate optical images with suitable spatial, temporal, and spectral resolution is expected to yield better estimation of health indicators [27], the accuracy is hindered by the fact that optical data are only sensitive to top of canopy in dense vegetated environments [151], and the spectra is highly influenced by the existence of NPV, BSC, and bare soil in sparsely vegetated areas [60,152,153] (Figure 2).

The presence of NPV, BSC, and bare soil affect spectral indices derived from optical remote sensing data and designed for the estimation of biophysical variables [153], such as LAI, green vegetation biomass, and fractions of Absorbed Photosynthetically Active Radiation (fAPAR) which are important attributes of EHA. LAI, fAPAR could be overestimated for ecosystems with randomly distributed sparse PV and NPV mixtures, while underestimated for dense mixtures due to the effects of NPV [153]. NPV accounts for similar amount of variations in spectral indices including NDVI and Modified Soil-adjusted Vegetation Index (MSAVI) [154] as green vegetation in semiarid mixed grassland. To reduce the influence of NPV, a hyperspectral index, litter-corrected ATSAVI (L-ATSAVI) was developed [140].

The presence of BSC can increase NDVI values by as high as 0.30 units in semiarid environments, which may result in overestimation of ecosystem productivity and misinterpretation of vegetation dynamics [60,70]. Comparatively, effects of bare soil on spectra have been extensively investigated since early 1970s [155,156]. The exerted influence on the spectra significantly affects NDVI and further affect LAI estimation [152] and green vegetation cover with the largest errors in grassland and shrubland areas [157]. Thus, many efforts were made to develop vegetation indices (e.g., a soil-adjusted vegetation index (SAVI, [152]) and a modified soil adjusted vegetation index (MSAVI, [158]) to minimize the soil brightness effects. Minimizing bare ground effects significantly improve the *r*^2^ value by 0.23 in estimating N in semiarid shrubland using HyMap hyperspectral data [78]. However, little research has been conducted to study the total effects of NPV, BSC and bare soil on vegetation indices and to evaluate the effects on the determination of single EHA attribute (e.g., LAI and productivity *etc.*) and to further identify their effects on a comprehensive EHA.

#### 4.4.2. Active Remote Sensing

Radar data have been widely used for structure and moisture content related ecosystem health indictors. However, the accuracy of estimation is dependent on many factors, such as characteristics of the instrument including frequency or wavelength, polarization, incident angle, look direction, and spatial resolution [159], and the properties of the land surface including surface roughness, and moisture content [160]. A discussion on those factors was provided in [59].

LiDAR data have also been used for retrieving ecosystem health indicators. The application of LiDAR data is basically based on the structure information LiDAR data can detect. The way LiDAR data are received (discrete return and full waveform LiDAR) and the footprint [161] may cause uncertainties in estimating health indicators. Full waveform LiDAR can provide more structure details than discrete LiDAR data. Generally, small footprint LiDAR has an advantage over detailed local mapping, and large footprint LiDAR is more suitable for investigating more interactions with multiple vertical structures and taking more complete ground sampling [161].

## 5. Conclusions/Outlook

A healthy ecosystem can sustainably provide the best quality ecological services to human beings, yet ecosystems worldwide have been impacted by climate change and anthropological activities. A comprehensive and dynamic ecosystem health assessment with the involvement of both ecologists and remote sensing specialists is needed.

The intrinsic temporal and spatial properties of remote sensing data provide an opportunity for developing a spatially explicit health ecosystem assessment and monitoring system. Currently, the issue of remote sensing ecosystem health is that the assessment is only based on a single indicator of one ecosystem attribute; however a comprehensive assessment should be a dynamic measurement of the three ecosystem attributes: vigor, organization, and resilience. The retrieval of different ecosystem health indicators may need diverse remote sensing data sources in terms of optical, radar, and LiDAR data, or optical data with different temporal, spectral, and spatial resolutions. To develop a comprehensive health ecosystem system with indicators of ecosystem vigor, organization, and resilience retrieved from different remote sensing imagery, currently one has to face the challenges of scale issue, transportability issue, data availability, and uncertainties in the estimation of indicators. Moreover, retrieval of some indicators, such as Non-photosynthetic Vegetation (NPV) biomass and Biological Soil Crust (BSC) cover, are still challenging.

As the technology in developing LiDAR, radar, and multi-angle optical sensors and methodologies for information retrieval improves, the uncertainties of estimation of health indicators are expected to decrease [162]. The integration of multi-sensoral data provides an opportunity to relieve the effects of scale issue. As the methods to develop the integrated models increase, the advantages of multiple sensors can be taken while minimizing disadvantages of each data source [162]. The multi-sensoral data may provide an opportunity to minimize the scale issues and reduce the uncertainties of health indicators. In addition, newly operational sensors and the upcoming sensors will also provide more opportunities for remote sensing of ecosystem health. Newly operational 10-day syntheses PROBA-V 333 m NDVI products since November 2013 are free for use to fill the gap of data discontinuity of SPOT 4 and SPOT vegetation-1 and vegetation-2 sensors [163]. In the near future, upcoming satellites, such as WorldView-3 satellite, Sentinel-2, the proposed Radarsat-2 constellation, and NovaSAR-S will provide more low cost opportunities for developing a spatially explicit ecosystem health assessment. As technological innovation in acquiring radar, LiDAR, and hyperspectral and multi-angle optical remote sensing data, and in algorithms and methods for retrieving information and integrating multi-sensoral data, developing a comprehensive, dynamic, and spatially explicit ecosystem health assessment and monitoring system will face fewer challenges.

The upcoming new sensors and upgraded technology on data processing will increase availability of remote sensing data and provide ecologists remote sensing products (e.g., biophysical parameter estimation, canopy structure, NPV biomass and BSC cover estimation *etc.*) with fewer uncertainties. This is expected to contribute to ecological studies by providing more accurate temporal and spatial details of ecological indicators, and thus aid ecologists to select more representative ecosystem health indictors. In turn, the progress of ecological studies in the regard of ecosystem health will provide feedbacks on the application of remote sensing data, which may facilitate the design of remote sensing sensors and the processing of remote sensing data. Through close collaboration, ecologists can obtain useful information on remote sensing data in terms of data attributes, application, limitation, and cost, while remote sensing specialists can acquire the most effective ecosystem health indicators in an efficient way. This will gradually bridge the research gap between ecologists and remote sensing specialists.
